# Modulating the proliferative and cytotoxic properties of patient-derived TIL by a synthetic immune niche of immobilized CCL21 and ICAM1

**DOI:** 10.3389/fonc.2023.1116328

**Published:** 2023-03-03

**Authors:** Sharon Yunger, Benjamin Geiger, Nir Friedman, Michal J. Besser, Shimrit Adutler-Lieber

**Affiliations:** ^1^ Ella Lemelbaum Institute for Immuno-Oncology, Sheba Medical Center, Ramat Gan, Israel; ^2^ Department of Immunology, The Weizmann Institute of Science, Rehovot, Israel; ^3^ Department of Immunology and Regenerative Biology, The Weizmann Institute of Science, Rehovot, Israel; ^4^ Department of Clinical Microbiology and Immunology, Sackler School of Medicine, Tel Aviv University, Tel Aviv, Israel; ^5^ Davidoff Center, Rabin Medical Center, Petah Tikva, Israel; ^6^ Advanced Technology Unit, Felsenstein Medical Research Center, Petah Tikva, Israel

**Keywords:** T-cells, tumor infiltrating lymphocytes (TIL), cancer immunotherapy, synthetic immune niche (SIN), adoptive cell therapy

## Abstract

A major challenge in developing an effective adoptive cancer immunotherapy is the *ex-vivo* generation of tumor-reactive cells in sufficient numbers and with enhanced cytotoxic potential. It was recently demonstrated that culturing of activated murine CD8+ T-cells on a “Synthetic Immune Niche” (SIN), consisting of immobilized CCL21 and ICAM-1, enhances T-cell expansion, increases their cytotoxicity against cultured cancer cells and suppresses tumor growth *in vivo*. In the study reported here, we have tested the effect of the CCL21+ICAM1 SIN, on the expansion and cytotoxic phenotype of Tumor Infiltrating Lymphocytes (TIL) from melanoma patients, following activation with immobilized anti-CD3/CD28 stimulation, or commercial activation beads. The majority of TIL tested, displayed higher expansion when cultured on the coated SIN compared to cells incubated on uncoated substrate and a lower frequency of TIM-3+CD8+ cells after stimulation with anti-CD3/CD28 beads. Comparable enhancement of TIL proliferation was obtained by the CCL21+ICAM1 SIN, in a clinical setting that included a 14-day rapid expansion procedure (REP). Co-incubation of post-REP TIL with matching target cancerous cells demonstrated increased IFNγ secretion beyond baseline in most of the TIL cultures, as well as a significant increase in granzyme B levels following activation on SIN. The SIN did not significantly alter the relative frequency of CD8/CD4 populations, as well as the expression of CD28, CD25, several exhaustion markers and the differentiation status of the expanded cells. These results demonstrate the potential capacity of the CCL21+ICAM1 SIN to reinforce TIL-based immunotherapy for cancer patients.

## 1 Introduction

Adoptive cancer immunotherapy, employing *ex vivo* expanded genetically modified or natural autologous T-cells, is a highly promising approach used for the treatment of cancer patients (1–5). Adoptive therapy approaches in cancer patients are mainly based on the administration of either Chimeric Antigen Receptor (CAR)-expressing T-cells (6, 7) or Tumor Infiltrating Lymphocytes (TIL) (8–10).

CAR T-cells are polyclonal T-cells, isolated from the patient’s peripheral blood, that are genetically engineered to express a CAR with the specificity of a monoclonal antibody, directed against a known tumor-associated antigen (TAA), along with a co-stimulatory signaling capacity (11, 12).

Conversely, TIL-based therapy uses the natural infiltration of T-cells into tumors, as indication for their tumor antigen recognition capacity, and their immunotherapeutic potential. TIL, that originally display insufficient proliferative and cytotoxic functionality under the suppressive conditions within the tumor, are isolated from the resected tumor tissue, and cultured *ex-vivo* under supportive restimulating conditions. Briefly, the *ex vivo* production process of TIL consists of two major stages (13–15): During the first stage, referred to as pre-Rapid Expansion Procedure (pre-REP), TIL cultures are being established, by the resection of a tumor biopsy, outgrowth of the tumor resident lymphocytes and their expansion, induced by interleukin-2 (IL-2) added to the culture medium. In the next stage - REP, established TIL cultures are massively expanded to treatment levels by addition of soluble anti-CD3 antibody, IL-2, and irradiated feeder cells. By the end of the REP, TIL cultures are administered back to the lympho-depleted cancer patient.

While there are unique advantages to TIL therapy over other adoptive cellular therapies, including its broad TCR diversity, relative safety and effective tumor homing, there are still major challenges to consider for its wide application, including varying clinical response rates in treating different patients and different types of tumors, variations in the REP expansion yields, unpredicted expansion of T-cell clones with different therapeutic capacities (16) and the risk that prolonged TIL expansion may result in impaired functionality, such as T-cell exhaustion or anergy (17–19).

Towards reinforcing adoptive cancer immunotherapy, a ‘Synthetic Immune Niche’ (SIN) strategy was recently developed by Adutler-Lieber et.al., based on the stimulation of T-cells by surfaces, coated with the chemokine C-C motif Ligand 21 (CCL21) and the Intercellular Adhesion Molecule 1 (ICAM-1) (20, 21).

CCL21, secreted by lymphatic stroma and endothelial cells (22) induces several processes critical to immune responses, including co-localization and recruitment of T-cells and dendritic cells (DCs) (23, 24); facilitating cell migration (25, 26); priming T-cells for synapse formation (27); and co-stimulation of naïve T-cell expansion and Th1 polarization (28–30). ICAM1 plays a key role in the formation of immune synapses and promotion of T cell activation, through binding to its integrin ligand, lymphocyte function-associated 1 (LFA1) (31, 32).

These factors are expected to act synergistically, as CCL21 increases LFA1 responsiveness to ICAM1, and mediates the arrest of motile lymphocytes on ICAM1 expressing DCs and endothelial cells, and their clustering with other T-cells (22, 33). Indeed, the developed SIN, consisting of a combination of immobilized CCL21 and ICAM-1, was shown to increase the expansion of activated murine CD4+ (2) and CD8+ (1) T-cells. Furthermore, culturing of OVA-specific CD8+ T-cells on this SIN elevates the cellular levels of granzyme B expressed by these cells and increases their efficiency in killing ovalbumin-expressing cultured cancer cells, as well as their tumor suppressive activity, *in vivo* (1).

In the present study we apply, for the first time, the CCL21+ICAM1 based SIN technology, on human TIL, derived from melanoma patients. We show here that SIN-treatment of anti CD3/CD28 activated cells display higher expansion rates than cells incubated on uncoated substrate. Comparable enhancement of TIL proliferation was also obtained when the SIN treatment was included in the rapid expansion process. Furthermore, testing of selected, SIN-treated, post-REP TIL cultures demonstrated increased IFNγ secretion and granzyme B expression levels, suggesting that the CCL21+ICAM1 SIN can reinforce the TIL-based immunotherapeutic approach.

## 2 Material and methods

### 2.1 Generation and expansion of TIL cultures

TIL samples were obtained from patients with metastatic melanoma enrolled to an open-label phase II ACT trial at the Sheba Medical Center (NCT00287131).

Tumor tissues were subjected to multi-stage processing that included mechanical fragmentation, tissue remnant culture (TRC), and enzymatic digestion (Digest) as previously described (15–17). The tumor was sliced with a scalpel into small pieces, about 1–3 mm^3^ in size. Enzymatic digestion of the pieces, with 100mg/ml collagenase (Sigma-Aldrich, Israel) and 1mg/ml dornase alfa (Pulmozyme, Genentech, South San Francisco, CA), was performed within 2 to 4 h after surgery for 2 h at 37°C or overnight at room temperature to obtain a single-cell suspension. Small tumor fragments (1–2 mm^3^ in size) or 1 × 10 E6 live nucleated cells, obtained by Digest or TRC, were plated per well in 24-well plates in 2 ml complete medium (CM) containing 10% human AB serum (Valley Biomedical, Winchester, VA), 2mM L-glutamine (Biological Industries, Israel), Pen/Strep (Biological Industries, Israel) and 3,000 IU/ml IL-2 (Chiron Novartis, New Jersey, USA) in RPMI 1640 medium (Gibco, Thermo Fisher Scientific, Waltham, MA). During the first week, non-adherent TIL cultures were transferred to a new 24-well plate and cultured separately from the adherent melanoma cells. Cells were split or fresh medium was added every 2 to 3 days, to maintain a cell concentration in the range of 0.5–2.0 × 10^6^ cells/ml. TIL cultures were established within 2 to 4 weeks.

### 2.2 Surface coating and functionalization with CCL21 and ICAM-1

Substrate functionalization was performed by overnight incubation with 5 µg/ml CCL21 and 50 µg/ml ICAM1 (R&D Systems, Minneapolis, MN, USA), in PBS.

### 2.3 Stimulation of TIL with anti CD3/CD28 antibodies

TIL cultures were stimulated for 7 days with anti-biotin MACSiBead Particles loaded with biotinylated CD3/CD28 antibodies (Miltenyi Biotec, Bergisch Gladbach, Germany). The preparation of the conjugated beads was carried out according to the manufacturer’s recommendation (activation/expansion kit, Miltenyi Biotec). Antibody-loaded anti-biotin MACSiBead™ were added to the TIL at a cell/bead ratio of 1:1. For plate-bound expansion, 24-well plates were coated with 0.3 ml PBS per well containing 2 μg/ml anti-CD3 (clone OKT-3; Miltenyi Biotech), 2 μg/ml anti-CD28 (clone: 15E8, Miltenyi Biotech) with or without addition of 5 µg/ml CCL21+ 50 µg/ml ICAM1 and incubated overnight at 4°C. The following day, plates were washed with PBS, 0.01 x10^6^ TIL in 2 ml CM medium with 3,000 IU/ml IL-2 were added per well for stimulation.

### 2.4 Rapid expansion procedure (REP) with CCL21 and ICAM-1 surface coating

The REP was initiated by stimulating 0.01-0.03 x10^6^ TIL with 30 ng/ml anti-CD3 antibodies (MACS GMP CD3 pure, clone OKT-3; Miltenyi Biotech, Germany), 3,000 IU/ml IL-2, and irradiated peripheral blood mononuclear cells from three non-related donors as feeder cells (50 Gy, TIL to feeder cells ratio = 1:100) in 50% CM, 50% AIM-V medium (Invitrogen, Thermo Fisher Scientific, Waltham, MA). REP was performed in 24-well plates. TIL seeding at days 0, 7, 11 started from range of 2-8 wells. CCL21+ICAM1 coating was prepared one day prior to the initiation or cell splitting (days -1, 6 and 10). TILs were cultured for 14 days and split on day 7 and 11 to maintain a cell concentration of 0.3-2.0x10^6^/well. Fold expansion was calculated by dividing the total number of cells at each time point by the number of cells at day 0.

### 2.5 Antibodies and flow cytometry

The following antibodies were used, in this study, for flow cytometry: Pacific blue-CD3 (clone SK7, BioLegend, San Diego, CA, United States), PECy7 or FITC-CD8 (clone HIT8a, BioLegend), FITC-PD-1 (clone EH12.2H7, BioLegend), FITC-TIM-3 (clone F38-2E2, BioLegend), FITC-LAG-3 (clone 11C3C65, BioLegend) APC-CD25 (clone BC96, BioLegend), APC-CD28 (clone CD28.2, BioLegend), APC-vio770-CD45RA (clone HI100, BioLegend) and PerCP-CCR7 (clone G043H7, BioLegend). TIL cultures were washed and re-suspended in cell-staining buffer (BioLegend). Cells were incubated for 30 min with the antibodies on ice, washed in buffer and measured using MACSQuant flow cytometer (Miltenyi Biotech).

For localization and quantification of granzyme B, cells were fixed (BLG420801, Biolegend), permeabilized (BLG421002, Biolegend), and stained with granzyme B-specific antibodies (BLG515406, BioLegend). For carboxy fluorescein succinimidyl ester (CFSE) cell proliferation assays, TIL cultures were stained before seeding at day 11 of the REP with 5 μM CFSE (Thermo Fisher Scientific) for 20 min at 37°C, according to the manufacturer’s instructions. Four days later cells were taken for flow cytometry analysis and their CFSE fluorescence intensity was determined. Samples were analyzed using FlowJo software (FlowJo LLC, Ashland, OR).

### 2.6 IFNγ ELISA

1x10^5^ TIL were co-cultured with autologous melanoma autologous melanoma cells in 96-well plates overnight at 1:1 effector-to-target ratio. Supernatant was collected and IFNγ levels were determined by ELISA (BioLegend) according to the manufacturer’s protocol.

### 2.7 Cell-mediated cytotoxicity assay

1x10^5^ TIL were co-cultured with autologous melanoma cells in 96-well plates overnight at a 1:1 effector-to-target ratio. Levels of lactate dehydrogenase (LDH) in the medium were determined by CytoTox 96 Non-Radioactive Cytotoxicity Assay (Promega, Madison, WI, Cat. no. G1780), carried out according to the manufacturer’s instructions. The luminescence of the samples was measured at 490 nm in a plate-reading luminometer (GloMax microplate luminescence reader, Promega Company, USA). Experiments were performed in triplicates.

### 2.8 Statistics

Significance of variation between groups was evaluated using a two-tailed Student’s t-test. Test for differences between proportions was performed using two-sided Fisher’s exact test with p ≤ 0.05 values considered as significant. (*p <.05, **p <.01, ***p <.001, ****p < .0001).

## 3 Results

### 3.1 Impact of CCL21+ICAM1 coated surface on TIL proliferation, following CD3/CD28 stimulation

**Figure 1:**
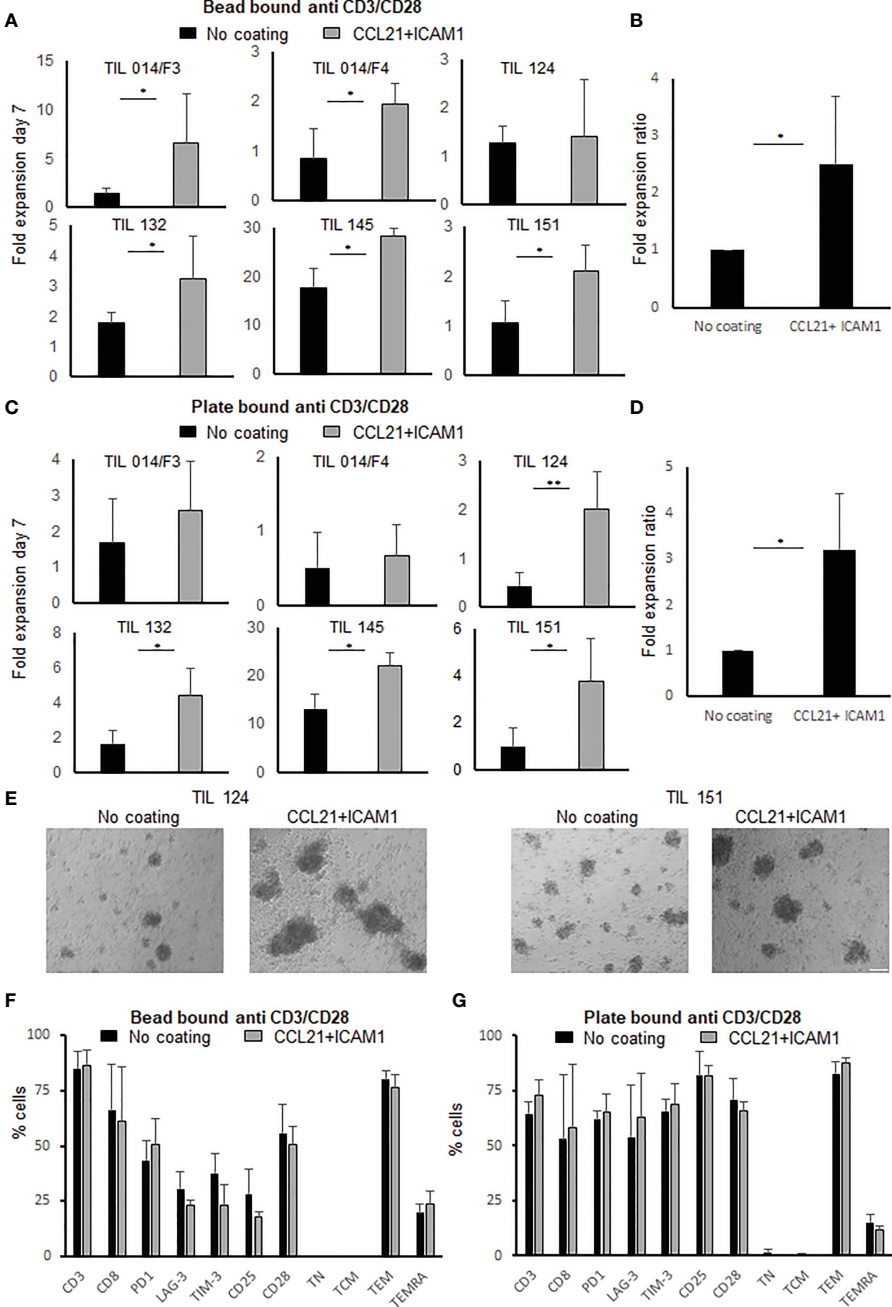
Fold expansion and phenotype analysis following anti-CD3/CD28 stimulation of TIL with or without exposure to SIN, consisting of CCL21+ICAM1 coated surface. **(A)** Fold expansion of TIL cultures (n=6) stimulated by anti-CD3/CD28 activation beads. Graph bars show comparison between TIL cultured on uncoated vs. CCL21+ICAM1 coated surfaces **(B)** Average fold expansion ratio between coated to uncoated TIL cultures (No coating=1), stimulated with activation beads (n=5). **(C)** Fold expansion of TIL cultures (n=6) stimulated by plate-bound anti-CD3/CD28 antibodies for 7 days. Graph bars show comparison between TIL cultured on uncoated vs. CCL21+ICAM1 coated surfaces. **(D)** Average fold expansion ratio between coated to uncoated TIL cultures (No coating=1), stimulated with plate-bound CD3/CD28 antibodies (n=4). **(E)** Morphology following 7-day plate-bound CD3/CD28 activation (scale bar = 100 µm). **(F, G)** Percentage of CD3, CD8, Distribution of surface markers: co-inhibitory: PD1, LAG-3, TIM-3, activation marker: CD25, co-stimulatory CD28 and TIL differentiation status: TN (naïve), CD3+CD45RA+CCR7+; TCM (central memory), CD3+CD45RA−CCR7+; TEM (effector memory), CD3+CD45RA−CCR7−; TEMRA (effector), CD3+CD45RA+CCR7− (n=4). (*p < .05, **p < .01).

To evaluate the effect of a CCL21+ICAM1 coated surface on the proliferative capacity of TIL cultures, the cells were seeded in 24-well plates, coated with CCL21+ICAM1 (“coated-TIL”) or a non-coated plates (“uncoated-TIL”) and activated by CD3/CD28 beads stimulation. Six TIL cultures were tested. TIL cultures were derived from five patients with metastatic melanoma (Pt. 014, 124, 132, 145 and 151) and two, independently established TIL cultures from the same patient (Pt. 014/F3 and 014/F4). Seven days following the initial stimulation, five out of the six TIL cultures showed higher fold expansion values following exposure to CCL21+ICAM1 (**Figure 1A**). In these five cultures, the expansion of coated-TIL was, on average, 2.5 ± 1.2-fold higher (range 1.6-4.6, p=.026) after 7 days, achieving an 8.4 ± 11.3-fold expansion for coated-TIL compared with 4.5 ± 7.3-fold for TIL cultured on uncoated surfaces (**Figure 1B**, normalized for uncoated TIL).

In addition, when stimulating TIL cultures with plate-bound anti-CD3 and anti-CD28 antibodies (instead of activation beads), four out of six TIL cultures exposed to CCL21+ICAM1 demonstrated higher fold expansion values (uncoated-TIL, 4.0 ± 6.1-fold; coated-TIL, 8.1 ± 9.4-fold, range 1.69-4.49) (**Figure 1C**). In these four cultures expansion of coated-TIL was on average 3.2 ± 1.2-fold higher proliferation (p = .012) over TIL cultured on uncoated dishes (**Figure 1D**, normalized for uncoated TIL). Moreover, microscopy-based monitoring demonstrated that SIN-stimulated TIL cultures tended to form larger cell aggregates than those incubated on non-coated plates (**Figure 1E**). By the end of the expansion, 77 ± 13% of the cells were CD3+ T cells. Independent of the anti-CD3/CD28 stimulation method (beads or immobilized), CCL21+ICAM1 exposure had no apparent impact on the CD4/CD8 subpopulation distribution, expression of the co-inhibitory molecules PD-1, LAG-3 and TIM-3, the co-stimulatory molecule CD28, the activation marker CD25 or the differentiation status of TIL (p values ≥.05) (**Figures 1F, G**). Moreover, there was no significant difference in the expression of sub-population markers within the CD4 or CD8 populations (p- values ≥.05) except for the inhibitory molecule TIM-3 and the activation marker CD25, which were significantly lower within the CD8+ T cells population following seven days of CD3/CD28 beads stimulation (TIM-3+CD8+, uncoated-TIL 31.2 ± 8.4%, coated-TIL 15.1 ± 7.4%, p=.029; CD25+CD8+ uncoated-TIL 12.1 ± 4.2%, coated-TIL 5.4 ± 2.8%, p=.036) (**Supplementary Tables 1, 2**). The gating strategy and representative flow cytometric plots are shown in **Suppl. Figure 1**.

### 3.2 CCL21+ICAM1 coated surfaces increased TIL proliferation during the rapid expansion procedure (REP)

**Figure 2:**
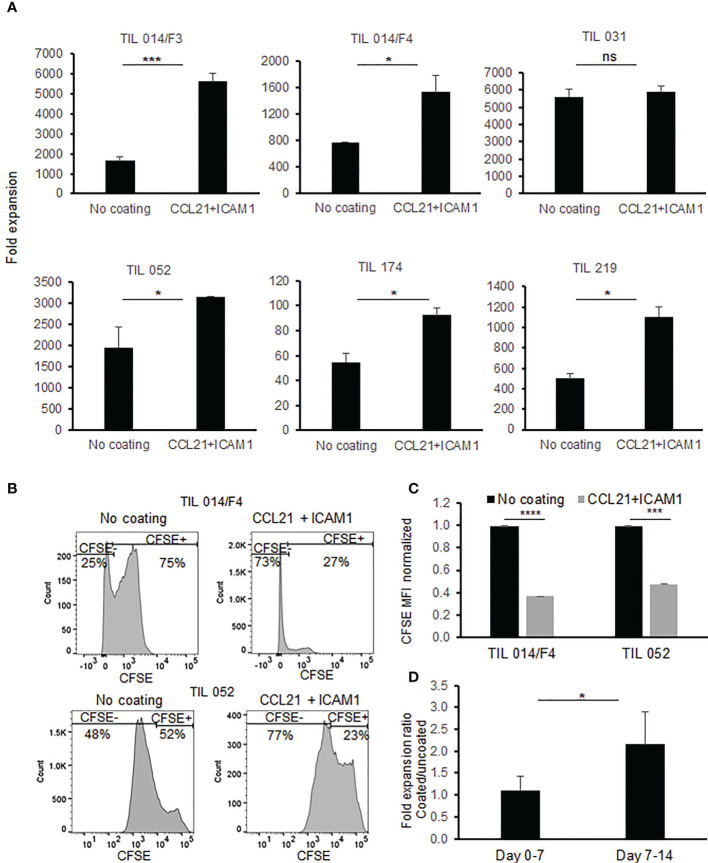
Comparison of TIL during REP with/without exposure to SIN, consisting of CCL21+ICAM1 coated surface. **(A)** Fold expansion on day 14 (n=6). All data are representatives of two experiments. The graphs show the mean ± SD values. **(B)** Histograms of CFSE staining **(C)** Bar graph shows mean fluorescence intensity of CFSE. TIL cultured on CCL21+ICAM1 coated surfaces normalized to the uncoated TIL (no coating= 1). **(D)** The average frequencies of fold expansions at day 0-7 and day 7-14, with or without incubation on CCL21+ICAM1 coated surface (n = 5). (*p < .05, ***p < .001, ****p < .0001, ns, not significant).

In the clinical setting, TIL cultures are expanded rapidly to large cell numbers by adding 40-50 Gy irradiated PBMC feeder cells, soluble anti-CD3 antibody and IL-2 to the cell culture. On day 14 of the *ex vivo* expansion, TIL cultures are harvested and prepared for the i.v. infusion. To test whether CCL21+ICAM1 coated surfaces improve the manufacturing process, small-scale REPs were initiated, closely resembling the clinical setting, using GMP-compliant reagents. Six TIL cultures obtained from five melanoma patients (Pt. 014, 052, 219, 031 and 174) were expanded according to the REP protocol for 14 days. As shown in **Figure 2A**, expansion was significantly increased on CCL21+ICAM1 coated surfaces by 2.3 ± 0.7-fold (p=.002) in 5 out of the 6 TIL cultures tested.

To confirm that CCL21+ICAM1 coating contributes to the enhanced expansion by increasing the proliferation rate, TIL cultures were stained with carboxyfluorescein succinimidyl ester (CFSE) dye on day 11 of the REP and analyzed on day 14. As shown in **Figures 2B, C**, a decrease in CFSE fluorescence intensity was observed in the SIN-stimulated TIL cultures indicating that the increased expansion is indeed attributable to elevated cell proliferation.

It was further shown that the major contribution of the CCL21+ICAM1 coated surface to the increased proliferation occurred during the second week of the REP (fold increase day 0-7 = 1.11 ± 0.32; fold increase day 7-14 = 2.15 ± 0.74; p = .020) (**Figure 2D**). The impact between day 7 to 11 and day 11 to 14 day was similar (fold increase day 7-11 = 1.61 ± 0.59; fold increase day 11-14: 1.39 ± 0.37; p=.511).

### 3.3 Increased expansion of TIL on CCL21+ICAM1 coated surface had no major impact on TIL phenotype

Analysis of pre-REP TIL cultures, derived from five patients, showed that 94.2 ± 7.3% of the pre-REP cells were CD3+ T cells.

**Figure 3:**
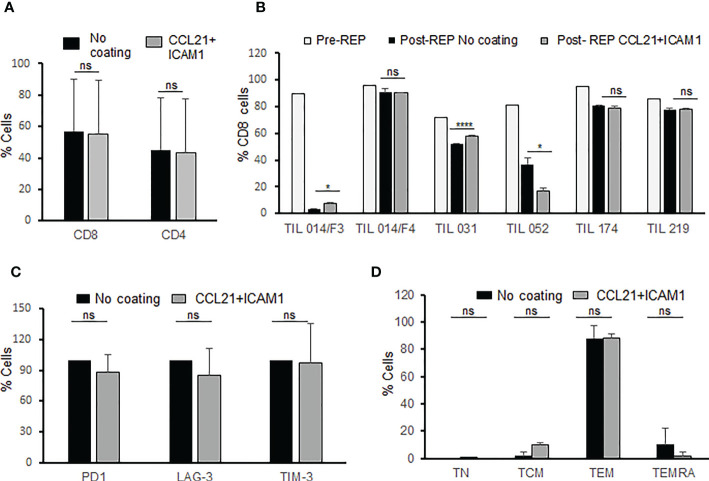
Phenotype analysis of post-REP TIL. **(A)** Frequency of CD8 and CD4 T cells for CCL21+ICAM1 coated and uncoated post-REP TIL cultures (n=6). **(B)** Frequency of CD8 T cells pre-REP and CCL21+ICAM1 coated and uncoated post-REP TIL cultures (n=6). **(C)** TIL cultures were characterized for expression of inhibitory molecules PD-1, LAG-3, TIM-3 (n = 4). The expression values of coated surface TIL were normalized to uncoated surface TIL (100%) **(D)** Distribution of differentiation subsets. TN (naive), CD3+CD45RA+CCR7+; TCM (central memory), CD3+CD45RA−CCR7+; TEM (effector memory), CD3+CD45RA−CCR7−; TEMRA (effector), CD3+CD45RA+CCR7−; (n=3). (*p < .05, ****p < .0001, ns, not significant).

To investigate the impact of CCL21+ICAM1 coated surfaces on the TIL phenotype, post-REP TIL cultures (n=6) were analyzed for CD4 and CD8 expression. CCL21+ICAM1 coating had no impact on the average frequency of CD4+ and CD8+ TIL (% CD4, coated-TIL = 45.1 ± 34.3%, uncoated-TIL 43.0% ± 33.0%, p = .917; %CD8 coated-TIL = 54.9 ± 34.8%, uncoated TIL = 57.0 ± 33.0%, p = .917) (**Figure 3A**).

As shown in **Figure 3B** (pre-REP versus post-REP), the rapid expansion procedure itself had a major impact on the percentage of CD8+ (and CD4+), however the effect of CCL21+ICAM1 coating was minor (**Figure 3B**) and on average insignificant (**Figure 3A**).

Four post-REP TIL cultures were further characterized for the expression of the inhibitory molecules PD-1, LAG-3, and TIM-3 on all CD3 T cells and on the CD4/CD8 T cell subpopulations. As shown in **Table 1**, expression of the inhibitory molecules was insignificantly different between coated and non-coated post-REP TIL cultures (p-values ≥.210) (**Figure 3C**).

**Table 1:** Phenotype analysis of post-REP TIL, cultured on CCL21+ICAM1 coated or uncoated surfaces.

	CD3+		PD1+		LAG-3+		TIM-3+	
TIL name	No coating	CCL21+ ICAM1	No coating	CCL21+ ICAM1	No coating	CCL21+ ICAM1	No coating	CCL21+ ICAM1
TIL 014/F3	94.4	92.1	53.8	56.5	55.3	28.5	24.5	12.9
TIL 014/F4	ND	ND	88.4	85.5	63.0	69.9	38.2	54.7
TIL 052	95.6	93.1	38.3	32.8	54.9	43.3	11.9	10.4
TIL 219	97.9	97.5	21.4	14.0	78.3	77.3	7.9	8.5
**Average**	**96.0**	**94.2**	**50.5**	**47.2**	**62.9**	**54.7**	**20.6**	**21.6**
SD	1.8	2.9	28.5	30.9	10.9	22.8	13.7	22.1
P value	0.424		0.881		0544		0.942
	**CD8+**		**PD1+ CD8+**		**LAG-3+ CD8+**		**TIM-3+ CD8+**	
TIL name	No coating	CCL21+ ICAM1	No coating	CCL21+ ICAM1	No coating	CCL21+ ICAM1	No coating	CCL21+ ICAM1
TIL 014/F3	3.4	7.3	2.5	5.5	3.2	7.7	0.2	0.5
TIL 014/F4	90.8	90.2	88.1	85.3	62.8	69.6	36.9	51.4
TIL 052	36.9	17.1	9.1	6.1	35.4	15.3	1.9	0.6
TIL 219	78.1	78.1	9.7	4.3	67.5	66.4	4.6	5.1
**Average**	**52.3**	**48.2**	**27.3**	**25.3**	**42.2**	**39.7**	**10.9**	**14.4**
SD	39.9	42.0	40.6	40.0	29.6	32.8	17.4	24.7
P value	0.892		0.945		0.914		0.824
	**CD4+**		**PD1+ CD4+**		**LAG-3+ CD4+**		**TIM-3+ CD4+**	
TIL name	No coating	CCL21+ ICAM1	No coating	CCL21+ ICAM1	No coating	CCL21+ ICAM1	No coating	CCL21+ ICAM1
TIL 014/F3	96.7	92.7	51.3	51.0	52.1	20.8	24.3	12.4
TIL 014/F4	9.2	9.8	0.3	0.2	0.2	0.3	1.3	3.3
TIL 052	63.1	82.9	29.2	26.7	19.5	28.0	9.9	9.7
TIL 219	21.9	21.9	11.7	9.7	10.8	10.9	3.3	3.3
**Average**	**47.7**	**51.8**	**23.1**	**21.9**	**20.7**	**15.0**	**9.7**	**7.2**
SD	39.9	42.0	22.2	22.3	22.4	12.0	10.4	4.6
P value	0.892		0.940		0.672		0.673

Averaged values are bold.

The differentiation status of three post-REP TIL cultures was defined based on the expression of CD45RA, in combination with CCR7. The majority of post-REP TIL, independently of whether they were cultured on a CCL21+ICAM1 surface or not, were CD45RA-CCR7-, effector memory T cells (TEM) (coated surfaces, TEM = 88.1 ± 3.4%; uncoated, TEM = 86.7 ± 9.6%; p = .830) (**Figure 3D**).

### 3.4 CCL21+ICAM1 coating increased the anti-tumor reactivity of TIL

To determine whether the CCL21+ICAM1 coated surfaces enhance anti-tumor reactivity, IFNγ secretion and granzyme B expression were determined after a co-culture of post-REP TIL cultures with or without autologous tumor target cells.

**Figure 4:**
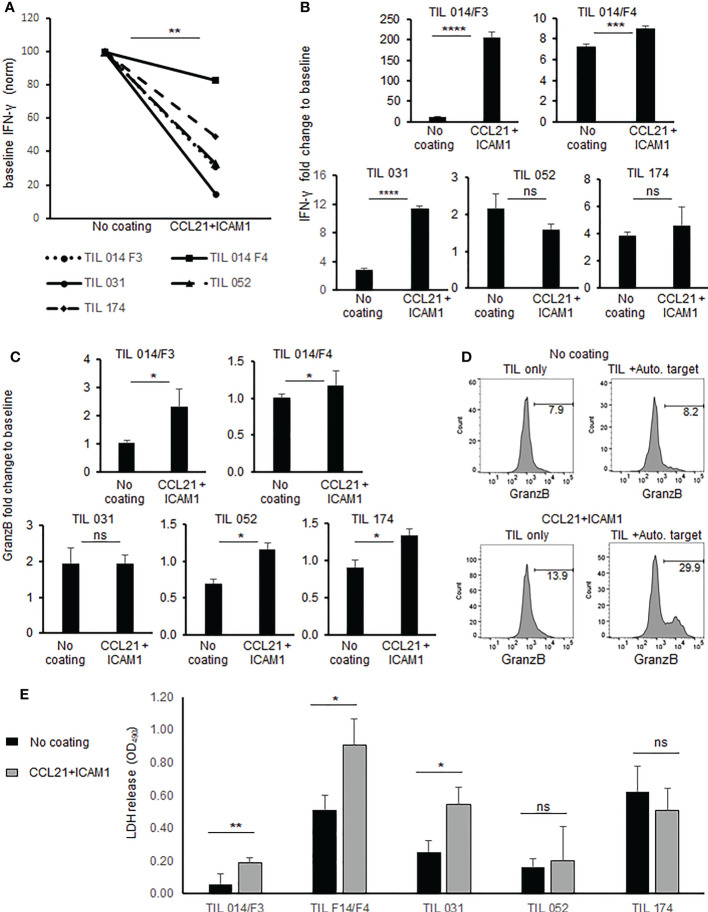
Anti-tumor reactivity and cytotoxicity of TIL cultured on SIN, consisting of CCL21+ICAM1 coated surfaces. **(A)** Connecting line histogram demonstrated different baseline (TIL only) of IFNγ secretion from CCL21+ICAM1 coated TIL or uncoated surfaces. **(B)** Fold change of IFNγ following co-cultures of TIL with autologous tumor versus TIL only. In each experiment, IFNγ concentration after co-culture was divided by the “TIL only” (= baseline) concentration and expressed as fold change. The bar plots show the mean fold change ± standard deviation (SD) of three independent experiments. **(C)** Fold change of MFI for granzyme B in CD8+ cells co-cultured with tumor cells. MFI values after co-culture were divided by the “TIL only” (= baseline) values and are presented as the mean ± standard deviation (SD) obtained in three independent experiments. **(D)** Representative FACS Histogram plots for granzyme **(B, E).** Cytotoxicity measured by LDH assay. Experiments were performed in triplicates wells. Results expressed as mean ± standard deviation (SD). (*p < .05, **p < .01, ***p < .001, ****p < .0001, ns, not significant).

Interestingly, the basal IFNγ level of TIL (without target cells) was significantly lower in cells exposed to coated surfaces compared with uncoated TIL (p = .0.001, **Figure 4A**). Three of five post-REP TIL cultures demonstrated significantly increased IFNγ secretion beyond baseline following exposure to the CCL21+ICAM1 coated surface (**Figure 4B**). Absolute IFNγ concentrations are shown in **Supplementary Table 3**.

Granzyme B expression was significantly increased (p ≤.05) in four out of five post REP TIL cultures which were expanded on coated surfaces (**Figure 4C**). Representative flow cytometric histograms are shown in **Figure 4D**. Direct cytotoxicity assay against autologous melanoma cells using LDH assay revealed in three of five post-REP TIL cultures significantly higher killing activity by TIL cultured on CCL21+ICAM1 coated surfaces, compared with uncoated TIL (**Figure 4E** and **Supp. Figure 2**).

## 4 Discussion

The primary motivation underlying the development of an *ex vivo* “synthetic immune niche”, that might improve the performance of therapeutic T-cells, is based on current experiments with diverse adoptive immunotherapies, whose effectiveness varies greatly between patients and tumor types (34). In our earlier studies we have shown that stimulation of murine CD4+ (1) or CD8+ T-cells (2) by a synthetic immune niche, consisting of immobilized ICAM-1 and CCL21, enhances the proliferation of both cell types, and increases the cytotoxic efficacy of CD8+ cells, both *ex vivo* and *in vivo*. These encouraging findings indicated that specific environmental stimulation can alter T-cell proliferation and activity, yet the experimental settings of these studies (35) (murine OT-1 and OT-2 systems), were very different from current clinical settings in humans. The primary goal of the current study was to determine the capacity of human CCL21+ICAM1 SIN to exert similar effects in a clinically relevant system.

The system we chose, namely TIL therapy, has earned great success in treating solid tumors (36), particularly metastatic melanoma (37–41), and does not require a prediction of the actual TAA, or the characteristics of its binding receptor. TIL cultures are naturally heterogeneous, potentially targeting a variety of tumor antigens, a feature that is particularly relevant to melanoma, which displays a high mutational load (42).

Moreover, the indication that the CCL21+ICAM1 SIN enhances expansion of T-cells, render the SIN approach highly suitable for TIL therapy, in which high numbers of cells are a favorable prognostic indicators for successful therapy in different tumor types (43–45) and specifically in foretelling the success of checkpoint therapies (46). The additional feature of this SIN, based on the results obtained using the murine system, namely, potentiation of the cytotoxic machinery by increasing granzyme B levels (1), is also highly relevant to TIL therapy, in which failed therapies are often attributed to exhaustion and anergy (21, 47).

The results presented herein strongly confirm that using the human SIN components CCL21+ICAM during anti-CD3/CD28 antibody stimulation significantly enhances patient-derived TIL expansion. In addition, CCL21+ICAM coated-TIL stimulated with anti-CD3/CD28 beads, demonstrated a significantly lower expression the co-inhibitory molecule TIM-3 and the activation marker CD25 on CD8+ T cells.

Also, when tested in a clinical setting that includes a 14-day rapid expansion procedure in the presence of irradiated feeder cells, anti-CD3 and IL-2, melanoma-derived TIL. Although the fold expansion values largely varied between different TIL cultures (1758 ± 2016-fold), in 5/6 TIL exposure to CCL21+ICAM SIN significantly increased the expansion rate (2900 ± 2425-fold).

Importantly, the increased expansion did not impact the expression of inhibitory molecules such as TIM-3, LAG-3 and PD-1 or the differentiation status of TIL. Moreover, post-REP TIL demonstrated increased IFNγ secretion beyond baseline in three of five cultures and a significant increase in granzyme B levels in four of five tested TIL cultures. Direct killing assay demonstrated enhanced killing of target cells following exposure of TIL to CCL21+ICAM1 surface

Further studies would be required to determine whether overexpression of CCL21+ICAM1 in the irradiated feeder cells could exert positive impact on TIL expansion during REP comparable to that of the substrate-immobilized SIN.

Given the results presented here, we propose that the CCL21+ICAM1 synthetic niche, which proved to enhance expansion in the murine OVA systems, and elevate the cytotoxic capacity of murine CD8 cells, is capable to reinforce human TIL, derived from melanoma patients. These results presented here strongly suggest that incorporation of SIN stimulation into the TIL expansion process might significantly improve the therapeutic performance of these cells.

## Data availability statement

The raw data supporting the conclusions of this article will be made available by the authors, without undue reservation.

## Author contributions

BG and MB contributed conception and design of the study. SY and SA-L performed experiments and acquired the data. SY wrote the first draft of the manuscript. BG, NF, and MB revised it critically for important intellectual content. All authors contributed to manuscript revision, read and approved the submitted version.
