# Supplementary material for: Modulating the proliferative and cytotoxic properties of patient-derived TIL by a synthetic immune niche of immobilized CCL21 and ICAM1
Source: Front Oncol. 2023 Mar 3;13:1116328. doi: 10.3389/fonc.2023.1116328 (PMC10020329; doi:10.3389/fonc.2023.1116328)
Supplement: Supplementary file 1 [file Presentation_1.pptx]

## Slide 1
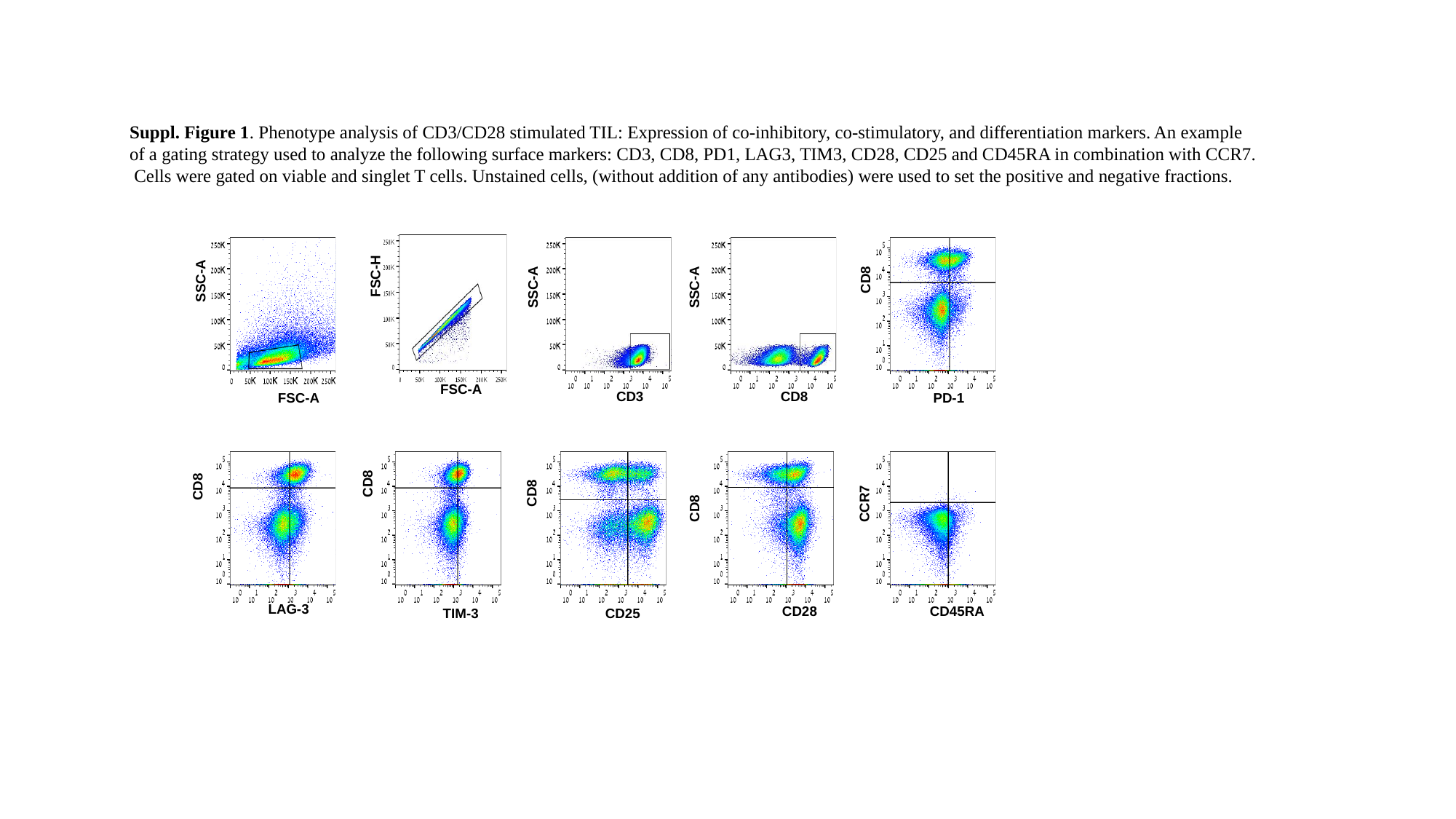

Suppl. Figure 1. Phenotype analysis of CD3/CD28 stimulated TIL: Expression of co-inhibitory, co-stimulatory, and differentiation markers. An example of a gating strategy used to analyze the following surface markers: CD3, CD8, PD1, LAG3, TIM3, CD28, CD25 and CD45RA in combination with CCR7. Cells were gated on viable and singlet T cells. Unstained cells, (without addition of any antibodies) were used to set the positive and negative fractions.
CD8
SSC-A
SSC-A
SSC-A
FSC-H
FSC-A
CD8
CD3
FSC-A
PD-1
CD8
CCR7
CD8
CD8
CD8
LAG-3
CD28
CD45RA
TIM-3
CD25
