# Supplementary material for: Modulating the proliferative and cytotoxic properties of patient-derived TIL by a synthetic immune niche of immobilized CCL21 and ICAM1
Source: Front Oncol. 2023 Mar 3;13:1116328. doi: 10.3389/fonc.2023.1116328 (PMC10020329; doi:10.3389/fonc.2023.1116328)
Supplement: Supplementary file 2 [file Presentation_2.pptx]

## Slide 1
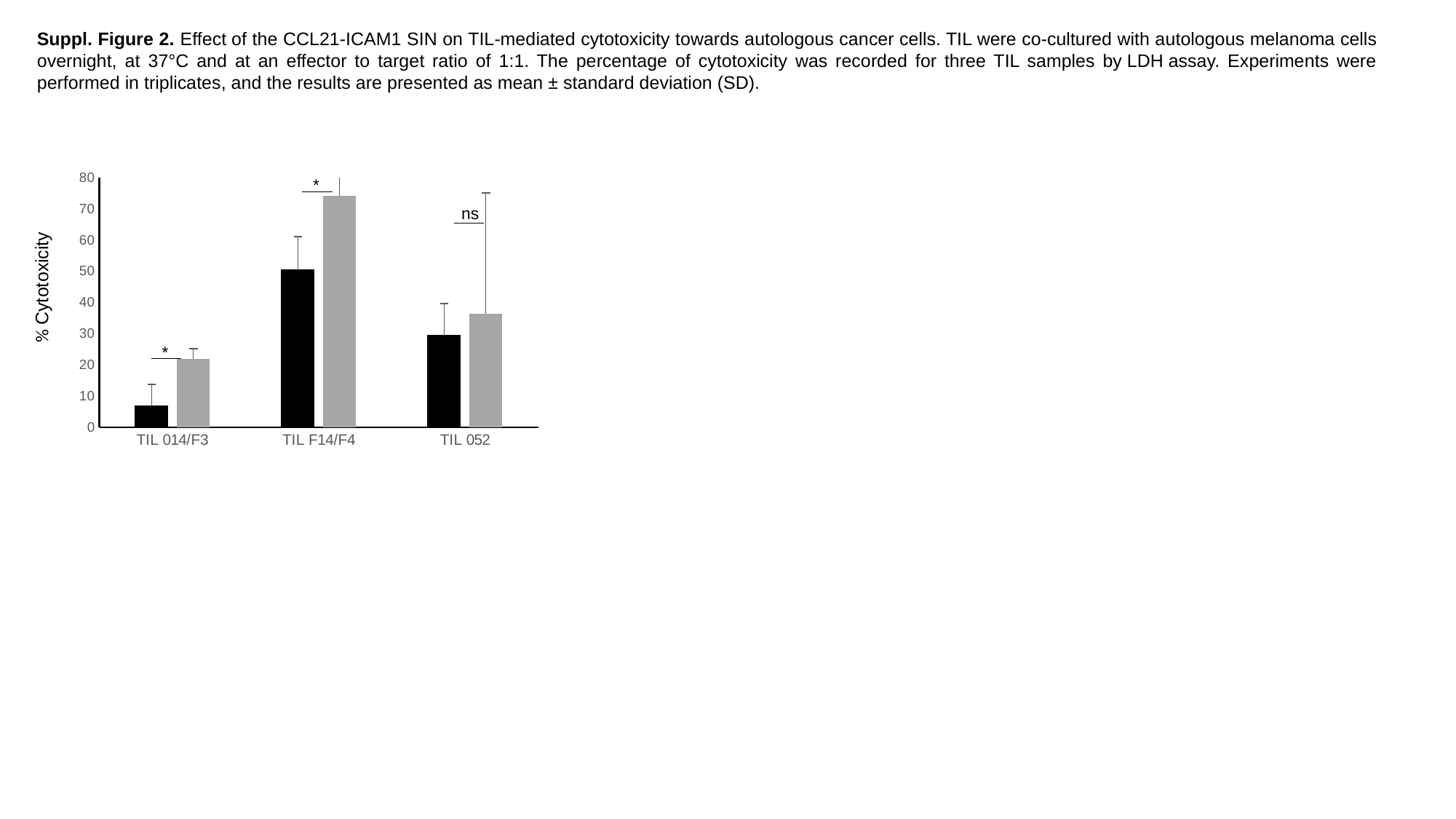

Suppl. Figure 2. Effect of the CCL21-ICAM1 SIN on TIL-mediated cytotoxicity towards autologous cancer cells. TIL were co-cultured with autologous melanoma cells overnight, at 37°C and at an effector to target ratio of 1:1. The percentage of cytotoxicity was recorded for three TIL samples by LDH assay. Experiments were performed in triplicates, and the results are presented as mean ± standard deviation (SD).
### Chart
| Category | avg | avg |
|---|---|---|
| TIL 014/F3 | 6.913661466457538 | 21.84847611194723 |
| TIL F14/F4 | 50.44972837603237 | 74.09156041645333 |
| TIL 052 | 29.443472095536396 | 36.45308703989468 |*
ns
% Cytotoxicity
*
