# Supplementary material for: Modulating the proliferative and cytotoxic properties of patient-derived TIL by a synthetic immune niche of immobilized CCL21 and ICAM1
Source: Front Oncol. 2023 Mar 3;13:1116328. doi: 10.3389/fonc.2023.1116328 (PMC10020329; doi:10.3389/fonc.2023.1116328)
Supplement: Supplementary file 3 [file Table_1.docx]

|  | **CD3+** | | **PD1+** | | **LAG-3+** | | **TIM-3+** | | **CD25+** | | **CD28+** | | |
| --- | --- | --- | --- | --- | --- | --- | --- | --- | --- | --- | --- | --- | --- |
| TIL name | No coating | CCL21+  ICAM1 | No coating | CCL21+  ICAM1 | No coating | CCL21+  ICAM1 | No coating | CCL21+  ICAM1 | No coating | CCL21+  ICAM1 | No coating | CCL21+  ICAM1 |  |
| TIL 014/F3 | 87.4 | 91.1 | 60.7 | 72.9 | 23.1 | 23.2 | 45.9 | 23.3 | 31.0 | 11.5 | 45.5 | 44.8 |  |
| TIL 124 | 83.9 | 77.7 | 43.3 | 52.4 | 19.0 | 19.1 | 29.9 | 9.4 | 8.4 | 6.8 | 63.6 | 45.6 |  |
| TIL 151 | 72.8 | 78.9 | 48.8 | 52.3 | 33.7 | 23.4 | 44.0 | 27.5 | 21.5 | 6.8 | 38.7 | 30.9 |  |
| TIL 145 | 95.8 | 96.4 | 21.2 | 24.9 | 46.3 | 27.3 | 30.8 | 32.8 | 52.8 | 45.7 | 75.6 | 81.4 |  |
| **Average** | **85.0** | **86.0** | **43.5** | **50.6** | **30.5** | **23.3** | **37.6** | **23.3** | **28.4** | **17.7** | **55.9** | **50.7** |  |
| SD | 7.6 | 7.4 | 8.9 | 11.9 | 7.6 | 2.4 | 8.7 | 9.5 | 11.3 | 2.7 | 12.9 | 8.3 |  |
| P value | 0.879 | | 0.600 | | 0.294 | | 0.070 | | 0.448 | | 0.718 | |  |
|  | **CD8+** | | **PD1+ CD8+** | | **LAG-3+ CD8+** | | **TIM-3+ CD8+** | | **CD25+ CD8+** | | **CD28+ CD8+** | | |
| TIL name | No coating | CCL21+ ICAM1 | No coating | CCL21+ ICAM1 | No coating | CCL21+ ICAM1 | No coating | CCL21+ ICAM1 | No coating | CCL21+ ICAM1 | No coating | CCL21+  ICAM1 |  |
| TIL 014/F3 | 58.6 | 59.3 | 39.8 | 46.6 | 18.6 | 14.7 | 36.2 | 14.7 | 14.1 | 3.5 | 17.9 | 13.3 |  |
| TIL 124 | 87.4 | 87.4 | 37.9 | 34.6 | 16.5 | 10.2 | 28.5 | 7.0 | 6.1 | 3.9 | 59.6 | 40.2 |  |
| TIL 151 | 77.7 | 68.6 | 38.1 | 36.4 | 27.3 | 17.9 | 39.4 | 25.0 | 15.6 | 4.6 | 29.1 | 25.0 |  |
| TIL 145 | 40.6 | 28.4 | 16.2 | 15.3 | 31.0 | 18.4 | 20.5 | 13.8 | 12.7 | 9.5 | 23.3 | 18.0 |  |
| **Average** | **66.1** | **60.9** | **33.0** | **33.2** | **23.4** | **15.3** | **31.2** | **15.1** | **12.1** | **5.4** | **32.5** | **24.1** |  |
| SD | 20.8 | 24.6 | 11.2 | 13.1 | 6.9 | 3.8 | 8.4 | 7.4 | 4.2 | 2.8 | 18.7 | 11.7 |  |
| P value | 0.760 | | 0.980 | | 0.087 | | 0.029 | | 0.036 | | 0.477 | |  |
|  | **CD4+** | | **PD1+ CD4+** | | **LAG-3+ CD4+** | | **TIM-3+ CD4+** | | **CD25+ CD4+** | | **CD28+ CD4+** | | |
| TIL name | No coating | CCL21+ ICAM1 | No coating | CCL21+ ICAM1 | No coating | CCL21+ ICAM1 | No coating | CCL21+ ICAM1 | No coating | CCL21+ ICAM1 | No coating | CCL21+ ICAM1 |  |
| TIL 014/F3 | 41.4 | 40.7 | 20.9 | 26.3 | 4.5 | 8.5 | 9.7 | 8.6 | 16.9 | 8.0 | 27.6 | 31.5 |  |
| TIL 124 | 12.6 | 12.6 | 5.4 | 17.8 | 2.5 | 8.9 | 1.4 | 2.4 | 2.3 | 2.8 | 4.0 | 5.4 |  |
| TIL 151 | 22.3 | 31.4 | 10.7 | 15.9 | 6.4 | 5.5 | 4.6 | 2.5 | 5.9 | 2.2 | 9.6 | 5.9 |  |
| TIL 145 | 59.4 | 71.6 | 5.0 | 9.6 | 15.3 | 8.9 | 10.3 | 19.0 | 40.1 | 36.2 | 52.3 | 63.4 |  |
| **Average** | **33.9** | **39.1** | **10.5** | **17.4** | **7.2** | **8.0** | **6.5** | **8.1** | **16.3** | **12.3** | **23.4** | **26.5** |  |
| SD | 20.8 | 24.6 | 7.4 | 6.9 | 5.6 | 1.6 | 4.2 | 7.8 | 17.0 | 16.1 | 21.8 | 27.4 |  |
| P value | 0.760 | | 0.222 | | 0.800 | | 0.726 | | 0.745 | | 0.863 | |  |

**Supplementary Table 1.** Phenotypic profile of TIL cultured on uncoated *vs* CCL21+ICAM1-coated surfaces, following CD3/CD28 beads stimulation. Cells were gated on viable, singlet CD3 T cells.
