# Supplementary material for: Modulating the proliferative and cytotoxic properties of patient-derived TIL by a synthetic immune niche of immobilized CCL21 and ICAM1
Source: Front Oncol. 2023 Mar 3;13:1116328. doi: 10.3389/fonc.2023.1116328 (PMC10020329; doi:10.3389/fonc.2023.1116328)
Supplement: Supplementary file 4 [file Table_2.docx]

|  | **CD3+** | | **PD1+** | | **LAG-3+** | | **TIM-3+** | | **CD25+** | | **CD28+** | | |
| --- | --- | --- | --- | --- | --- | --- | --- | --- | --- | --- | --- | --- | --- |
| TIL name | No coating | CCL21+  ICAM1 | No coating | CCL21+  ICAM1 | No coating | CCL21+  ICAM1 | No coating | CCL21+  ICAM1 | No coating | CCL21+  ICAM1 | No coating | CCL21+  ICAM1 |  |
| TIL 014/F3 | 51.4 | 76.6 | 70.7 | 81.7 | 25.9 | 41.3 | 75.2 | 79.4 | 88.2 | 84.4 | 81.6 | 75.7 |  |
| TIL 124 | 59.9 | 65.7 | 67.5 | 65.8 | 65.6 | 67.7 | 65.1 | 62.1 | 77.8 | 75.0 | 72.4 | 69.8 |  |
| TIL 151 | 61.6 | 78.4 | 75.4 | 76.3 | 67.7 | 80.9 | 65.9 | 78.0 | 66.4 | 75.7 | 63.1 | 67.6 |  |
| TIL 145 | 85.7 | 71.9 | 33.4 | 37.7 | 56.5 | 61.3 | 56.2 | 55.2 | 95.8 | 90.8 | 67.1 | 50.7 |  |
| **Average** | **64.7** | **73.2** | **61.8** | **65.4** | **53.9** | **62.8** | **65.6** | **68.7** | **82.0** | **81.5** | **71.0** | **66.0** |  |
| SD | 5.5 | 6.9 | 4.0 | 8.1 | 23.6 | 20.2 | 5.6 | 9.6 | 10.9 | 5.2 | 9.3 | 4.2 |  |
| P value | 0.323 | | 0.800 | | 0.511 | | 0.680 | | 0.942 | | 0.475 | |  |
|  | **CD8+** | | **PD1+ CD8+** | | **LAG-3+ CD8+** | | **TIM-3+ CD8+** | | **CD25+ CD8+** | | **CD28+ CD8+** | | |
| TIL name | No coating | CCL21+ ICAM1 | No coating | CCL21+ ICAM1 | No coating | CCL21+ ICAM1 | No coating | CCL21+ ICAM1 | No coating | CCL21+ ICAM1 | No coating | CCL21+  ICAM1 |  |
| TIL 014/F3 | 19.0 | 33.4 | 13.7 | 26.8 | 10.9 | 23.9 | 13.6 | 27.0 | 9.9 | 17.7 | 11.7 | 18.7 |  |
| TIL 124 | 84.6 | 85.1 | 51.9 | 55.5 | 59.4 | 62.6 | 58.0 | 58.8 | 66.2 | 63.2 | 68.9 | 64.1 |  |
| TIL 151 | 68.6 | 81.4 | 52.1 | 64.4 | 52.1 | 71.6 | 52.7 | 71.3 | 33.6 | 55.7 | 40.5 | 55.8 |  |
| TIL 145 | 40.6 | 33.0 | 10.8 | 20.5 | 17.1 | 29.1 | 15.7 | 24.7 | 19.1 | 28.8 | 10.7 | 10.5 |  |
| **Average** | **53.2** | **58.2** | **32.1** | **41.8** | **34.9** | **46.8** | **35.0** | **45.5** | **32.2** | **41.4** | **33.0** | **37.3** |  |
| SD | 29.2 | 28.9 | 23.0 | 21.4 | 24.4 | 23.8 | 23.6 | 23.2 | 24.7 | 21.6 | 27.7 | 26.6 |  |
| P value | 0.815 | | 0.561 | | 0.511 | | 0.551 | | 0.597 | | 0.829 | |  |
|  | **CD4+** | | **PD1+ CD4+** | | **LAG-3+ CD4+** | | **TIM-3+ CD4+** | | **CD25+ CD4+** | | **CD28+ CD4+** | | |
| TIL name | No coating | CCL21+ ICAM1 | No coating | CCL21+ ICAM1 | No coating | CCL21+ ICAM1 | No coating | CCL21+ ICAM1 | No coating | CCL21+ ICAM1 | No coating | CCL21+ ICAM1 |  |
| TIL 014/F3 | 81.0 | 66.6 | 57.0 | 54.9 | 15.0 | 17.4 | 61.6 | 52.4 | 78.3 | 66.7 | 69.9 | 57.0 |  |
| TIL 124 | 15.4 | 14.9 | 15.6 | 10.3 | 6.2 | 5.1 | 7.1 | 3.3 | 11.6 | 11.8 | 3.5 | 5.7 |  |
| TIL 151 | 31.4 | 18.6 | 23.3 | 11.9 | 15.6 | 9.3 | 13.2 | 6.7 | 32.8 | 20.0 | 22.6 | 11.8 |  |
| TIL 145 | 59.4 | 67.0 | 22.6 | 17.2 | 39.4 | 32.2 | 40.5 | 30.5 | 76.7 | 62.0 | 56.4 | 40.2 |  |
| **Average** | **46.8** | **41.8** | **29.6** | **23.6** | **19.1** | **16.0** | **30.6** | **23.2** | **49.9** | **40.1** | **38.1** | **28.7** |  |
| SD | 29.2 | 28.9 | 18.6 | 21.1 | 14.2 | 11.9 | 25.3 | 22.9 | 33.1 | 28.2 | 30.5 | 24.1 |  |
| P value | 0.815 | | 0.682 | | 0.753 | | 0.681 | | 0.670 | | 0.645 | |  |

**Supplementary Table 2**. Phenotypic profile of TIL cultured on uncoated *vs* CCL21+ICAM1-coated surfaces, following stimulation with plate-bound anti CD3 and anti CD28 antibodies. Cells were gated on viable, singlet CD3 T cells.
