# Supplementary material for: Modulating the proliferative and cytotoxic properties of patient-derived TIL by a synthetic immune niche of immobilized CCL21 and ICAM1
Source: Front Oncol. 2023 Mar 3;13:1116328. doi: 10.3389/fonc.2023.1116328 (PMC10020329; doi:10.3389/fonc.2023.1116328)
Supplement: Supplementary file 5 [file Table_3.docx]

**Supplementary Table 3:** IFN-γ concentrations (left) or percentages of CD8+ cells expressing Granzyme B (right) were determined after co-culture of TIL with autologous melanoma tumors with CCL21+ICAM1 coating or without coating.

|  | **IFN-γ (pg/ml)** | | | | **CD8+ Granzyme B (%)** | | | |
| --- | --- | --- | --- | --- | --- | --- | --- | --- |
|  | **No coating** | | **CCL21+ ICAM1** | | **No coating** | | **CCL21+ ICAM1** | |
|  | **TIL only** | **TIL+Target** | **TIL only** | **TIL+Target** | **TIL only** | **TIL+Target** | **TIL only** | **TIL+Target** |
| **TIL14/F3** | 110.0±42.3 | 1343.9±203.8 | 33.9±1.4 | 6950.8±502.0 | 7.3±1.0 | 9.0+1.1 | 15.1±1.1 | 28.3±2.4 |
| **TIL014/F4** | 628.0±131.7 | 4592.7±110.4 | 521.3±68.6 | 4721.3±95.1 | 18.6±3.3 | 18.6±1.9 | 27.4±1.4 | 30.0±0.4 |
| **TIL 031** | 491.0±297.8 | 1399.5±275.7 | 70.7±42.4 | 803.2±137.6 | 10.4±1.5 | 25.7±4.0 | 5.0±1.0 | 14.4±0.6 |
| **TIL 052** | 251.7±45.1 | 543.9±97.7 | 82.3±7.4 | 131.1±13.2 | 40.0±3.9 | 40.1±5.4 | 7.4±1.9 | 10.3±2.3 |
| **TIL 174** | 296.7±33.3 | 1142.0±71.1 | 146.0±5.3 | 673.3±193.1 | 12.3±2.3 | 13.4±2.9 | 5.2±1.1 | 8.4±1.2 |
